# Genetic Characterization of *bla*_CTX–M–55_ -Bearing Plasmids Harbored by Food-Borne Cephalosporin-Resistant *Vibrio parahaemolyticus* Strains in China

**DOI:** 10.3389/fmicb.2019.01338

**Published:** 2019-06-18

**Authors:** Zhiwei Zheng, Ruichao Li, Lianwei Ye, Edward Wai-chi Chan, Xiaodong Xia, Sheng Chen

**Affiliations:** ^1^College of Food Science and Engineering, Northwest A&F University, Yangling, China; ^2^Shenzhen Key Laboratory for Food Biological Safety Control, Food Safety and Technology Research Centre, The Hong Kong PolyU Shenzhen Research Institute, Shenzhen, China; ^3^State Key Laboratory of Chirosciences, Department of Applied Biology and Chemical Technology, The Hong Kong Polytechnic University, Kowloon, Hong Kong; ^4^College of Veterinary Medicine, Yangzhou University, Yangzhou, China

**Keywords:** *bla*_CTX-M-55_, *V. parahaemolyticus*, conjugative plasmid, cephalosporin resistance, IncC plasmid

## Abstract

This study aims to investigate and compare the complete nucleotide sequences of the multidrug resistance plasmids pVb0267 and pVb0499, which were recovered from foodborne *Vibrio parahaemolyticus* isolates, and analyze the genetic environment of *bla_CTX–M–55_* to provide insight into the dissemination mechanisms of this resistance element. Analysis of the sequences of plasmids pVb0267 (166,467 bp) and pVb0499 (192,739 bp) revealed that the backbones of these two plasmids exhibited a high degree of similarity with pR148, a recognized type 1a IncC plasmid recovered from *Aeromonas hydrophila* (99% identity). The resistance genes, found in both plasmids, included *qacH, aadB, arr2, bla*_OXA–10_*, aadA1, sul1, tet*(A), and *bla*_CTX–M–55_, which were mostly arranged in a specific region designated ARI-A. Plasmid pVb0499 was found to possess a larger size of ARI-A than pVb0267, which lacked a *mer* determination region, a *qnr* A segment, an *aacC3* gene and several mobility-encoding genes. Comparative analysis of resistance island (RI) of these plasmids and others revealed the potential evolution route of these RI sequences. In conclusion, plasmids harboring the *bla*_CTX–M–55_ gene has been recovered in *Vibrio parahaemolyticus* strains of food origin. It is alarming to find that IncC plasmids harboring resistance islands are disseminating in aquatic bacterial strains. The continuous evolution of resistance genes in conjugative plasmid in aquatic bacteria could be due to bacterial adaption to aquaculture environment, where antibiotics were increasingly used.

## Introduction

*Vibrio parahaemolyticus* is a gram-negative, halophilic, mesophilic, rod-shaped human pathogen that naturally occurs in the marine or estuarine environment (Lüdeke et al., 2015). Although *V*. *parahaemolyticus* is frequently isolated from a variety of seafood, such as shrimp, oyster, and fish, most isolates are non-pathogenic to human (Nishibuchi and Kaper, 1985). However strains carrying the *tdh* and *trh* genes can cause acute human gastroenteritis with the symptoms of headache, abdominal pain, and diarrhea, and in rare cases, wound infection and septicemia (Nishibuchi and Kaper, 1985; Zhang and Austin, 2005; Broberg et al., 2011). Seafood has gained enormous popularity leading to high demand, which has resulted in steady expansion of the Asian aquaculture industry. As a major vehicle of transmission of foodborne bacteria, increasing consumption of seafood could also lead to increase in foodborne illnesses in human. *V*. *parahaemolyticus* has become one of the most common causative agents of foodborne diseases in China in recent years, with the majority of infection cases being linked to consumption of contaminated seafood (Chen et al., 2010; Peng et al., 2010).

With the development and expansion of aquaculture practices, the health of aquatic animal has been under constant challenge (Bondad-Reantaso et al., 2005). To prevent and treat bacterial infections, a broad range of antibiotics such as oxytetracycline, tetracycline, quinolones, sulphonamides, and trimethoprim has been permitted for usage in the Asian aquaculture industry (Yano et al., 2014). The extensive use of antibiotics in aquaculture has resulted the emergence of antibiotic resistant strains in the environment. Besides, an increasing trend of resistance to front line antibiotics such as ciprofloxacin and ceftriaxone in *V*. *parahaemolyticus* has been reported in China and other parts of the world. Different β-lactamase-encoding genes such as *bla*_CMY–2_*, bla*_PER–1__,_ and *bla*_VEB–2_, which mediate cephalosporin resistance, have been detectable in *V*. *parahaemolyticus* (Li et al., 2015a, 2015b; Briet et al., 2018). Nevertheless, genes in the CTX-M family, commonly harbored by cephalosporin resistant bacteria (Naseer and Sundsfjord, 2011), remain rarely detectable in *V*. *parahaemolyticus* or other *Vibrio* spp. (Zheng et al., 2018). The *bla*_CTX–M–55_ gene belongs to the CTX-M-1 group and differs from *bla*_CTX–M–15_ by a single amino acid substitution of Val-77-Ala. The *bla*_CTX–M–55_ gene was first detected in clinical strains of *E. coli* and *K. pneumoniae* isolated in Thailand in 2005 (Kiratisin et al., 2007), and was subsequently found in *Salmonella* spp. in China, the United States, Korea, and Switzerland (Sjölund-Karlsson et al., 2011; Dahmen et al., 2013). A major epidemiological feature of Extended Spectrum Beta-Lactamases (ESBL)-producing strains is that the *bla*_CTX–M–55_ gene has emerged as a dominant resistance genotype among these strains. The prevalence rate of clinical isolates containing *bla*_CTX–M–55_ has exceeded those carrying *bla*_CTX–M–15_ in China, becoming the second most common resistance genotype among ESBL-producing strains (Zhang et al., 2014). Detection of *bla*_CTX–M–55_ is an alarming signal that depicts successful transmission of this important ESBL gene from *Enterobacteriaceae* to marine organisms. Continuous surveillance of this evolution process is therefore warranted.

Generally, *Vibrio* spp. are known to be susceptible to most clinically used antibiotics (Shaw et al., 2014; Letchumanan et al., 2015a). It is noted that most of the antibiotic resistance determinants are located in the plasmid, which is the most important mediator that facilitate the transfer of antibiotic resistant genes (Okamoto et al., 2009; Letchumanan et al., 2015b). IncC group plasmids have been identified in various species, indicating that such plasmids have been disseminated to, and accommodated by a broad range of bacterial hosts since they were first described among multidrug resistant *Aeromonas hydrophila* causing disease in cultured fish in the 1970s (Fricke et al., 2009; Sekizuka et al., 2011; Harmer and Hall, 2015; Li et al., 2015a). Moreover, their role in dissemination of ESBL and carbapenemase genes has attracted attention of researchers (Fricke et al., 2009; Sekizuka et al., 2011; Li et al., 2015a). IncA and IncC plasmids were grouped as the “A-C complex” at the beginning due to structural similarities and the strong entry exclusion (Hedges, 1974). Subsequently the term IncA/C has been used frequently to describe these two kinds of plasmid (Couturier et al., 1988; Harmer and Hall, 2015). Recently IncA and IncC plasmids have been proven to be compatible in the same bacterial strain and exhibited separate evolutionary routes and significant nucleotide divergence between their backbones (Ambrose et al., 2018). Thus, IncA/C is not used together anymore to describe plasmids. Analysis of the complete sequences of IncC plasmids to date has revealed that they are mostly large, ranging in size from 110 to 200 kb, self-mobilizable and confer resistance to a broad range of antibiotics. Upon acquisition by enterobacteria in both human and animals, IncC plasmids have apparently rapidly evolved and a number of lineages have been identified subsequently, with each carrying unique resistance genes (Harmer and Hall, 2015). In this work, we described the isolation and characterization of CTX-M-55 producing *V*. *parahaemolyticus* strains of food origin and depicted the evolution of IncC types of plasmids that harbored *bla*_CTX–M–55_.

## Materials and Methods

### Bacterial Isolation and Identification

The *V*. *parahaemolyticus* strains tested in this work were isolated from shrimp samples collected from a free market and two supermarkets located in the Nanshan district of Shenzhen, China, during the period August to October 2015. Samples were processed as previously described (Pinto et al., 2011). Briefly, 20 g of shrimp samples was homogenized with 50 mL of sterile saline. One milliliter saline homogenate was then added to 9 mL alkaline peptone water (APW) for enrichment of the strains at 37°C overnight. After incubation, the enrichment broths were streaked onto thiosulfate-citrate-bile salts-sucrose (TCBS) agar plates and incubated at 37°C for 18–24 h. At least three typical colonies of *V. parahaemolyticus* were isolated from each plate and subjected to identification by multiplex PCR assays and DNA sequencing (Kim et al., 2015). The genetic identity of the isolates was further confirmed by MALDI-TOF (Bruker).

### Antimicrobial Susceptibility Tests and Conjugation Assay

The *V. parahaemolyticus* isolates were subjected to antimicrobial susceptibility tests, using the standard agar dilution method described by the Clinical and Laboratory Standards Institute (CLSI) (Jorgensen, 2015). *Escherichia coli* strain ATCC 25922 was included as the quality control strain. Interpretation of results was according to the latest CLSI breakpoints. The minimal inhibitory concentrations (MICs)of eleven antibiotics were tested on the *V. parahaemolyticus* isolates: ceftriaxone, cefotaxime, amoxocillin-clavulanic acid, ampicillin, tetracycline, amikacin, gentamicin, ciprofloxacin, nalidixic acid, chloramphenicol, and sulfamethoxazole-trimethoprim.

Conjugation by filter mating was performed to test the transferability of mobile resistance elements between the cephalosporin-resistant *V. parahaemolyticus* isolates and the azide-resistant *E. coli* strain J53. Both the donor and recipient strains were cultured to exponential phase in LB broth, 300 μL of each culture were collected and mixed together. The mixture was spotted on a filter membrane that was placed on an LB agar plate, and then incubated for mating at 37°C for 12–18 h. Bacteria were washed from filter membrane and spread onto an Eosin methylene blue (EMB) agar plate containing 2 mg/L of cefotaxime to select transconjugants, as only *E. coli* strains could grow in EMB, while *V. parahaemolyticus* strains could not. The species of transconjugants was confirmed by MALDI-TOF. The MICs for the transconjugants were also determined according to the aforementioned method.

### Molecular Detection of β-Lactamase Genes

The cephalosporin-resistant isolates and the corresponding transconjugants were screened for carriage of known β-lactamase genes using a previously described multiplex PCR assay (Dallenne et al., 2010). PCR was carried out in 20 μL of reaction mixture which contained DNA template (2 μL), 1 × PCR buffer (TaKaRa, Japan), 200 mM of each deoxynucleotide triphosphate, a variable concentration of specific-group primers (Supplementary Table S3) and 1 U of Taq polymerase (TaKaRa, Japan). Amplification was performed with the following conditions: initial denaturation at 94°C for 10 min; 30 cycles of 94°C for 40 s, 60°C for 40 s and 72°C for 1 min; and a final elongation step at 72°C for 7 min. For the carbapenemase gene multiplex PCR assays, the annealing temperature was optimal at 55°C for amplification of *bla*_VIM_, *bla*_IMP__,_ and *bla*_KPC_ genes, and optimal at 57°C for amplification of *bla*_GES_ and *bla*_OXA–48_ genes. All PCR products were visualized by using 1.5% agarose gel and sequenced for further confirmation.

### Molecular Typing by PFGE and Southern Blot Analysis

To evaluate the genetic relationship of the isolates, pulsed-field gel electrophoresis (PFGE) was performed. Specifically, XbaI-digested genomic DNA was prepared according to the manufacturer’s instructions and restricted fragments were separated on a CHEF MAPPER system (Bio-Rad, United States) for 18 h at 14°C with run conditions of 6 V/cm and switch times from 6.76 to 35.38 s. The results of PFGE were interpreted as recommended by Tenover et al. (1995). To estimate the size and location of *bla*_CTX–M–55_ in the parental strain and the transconjugant, genomic DNA was digested with S1-nuclease (TaKaRa, Japan), followed by pulsed-field gel electrophoresis (S1-PFGE) as described above. The DNA fragments were then transferred to a positive-charged nylon membrane. Southern hybridization was carried out in accordance to the manufacturer’s instructions for digoxigenin (DIG)-High Prime DNA labeling, using a digoxigenin-labeled *bla*_CTX–M–55_ specific probe. Finally, the DNA fragments were detected using an NBT/BCIP color detection kit (Roche, Germany) (Hui et al., 2007). The XbaI-digested DNA of *Salmonella enterica* serotype Braenderup H9812 was electrophoresed as the size marker.

S1 nuclease-PFGE and Southern blotting were performed to estimate the size of plasmids in the parental strains and transconjugants. Plasmid DNA electrophoresis, followed by transfer to nylon membrane, were performed according to standard methods (Li et al., 2015a). Southern blot hybridization and probe detection were carried out in accordance with the manufacturer’s instructions of the digoxigenin (DIG)-High Prime DNA labeling and detection starter kit II (Roche Diagnostics), using a digoxigenin-labeled *bla*_CTX–M–55_ probe.

### Plasmid Analysis

Plasmids of the *bla*_CTX–M–55_-positive transconjugants were extracted using the Plasmid Extraction Kit (Qiagen, Hilden, Germany). The plasmids recovered from transconjugants were sequenced by the Illumina NextSeq 500 platform and the MinION sequencing platform. The method used to perform MinION sequencing was reported previously; Unicycler was used to perform hybrid assembly (Wick et al., 2017; Li et al., 2018). The completed plasmid sequence was confirmed by PCR and then annotated with the RAST tool and the NCBI Prokaryotic Genome Annotation Pipeline. BLAST tools were used for the comparative analysis of plasmid sequence and ARI-A sequences.

### Nucleotide Sequence Accession Number

The complete nucleotide sequences of pVb0267 and pVb0499 have been submitted to GenBank and were assigned the accession numbers of MF627444 and MF627445, respectively.

## Results

### Phenotypic and Genotypic Characteristics of Cephalosporin Resistance in *V. parahaemolyticus*

**FIGURE 1:**
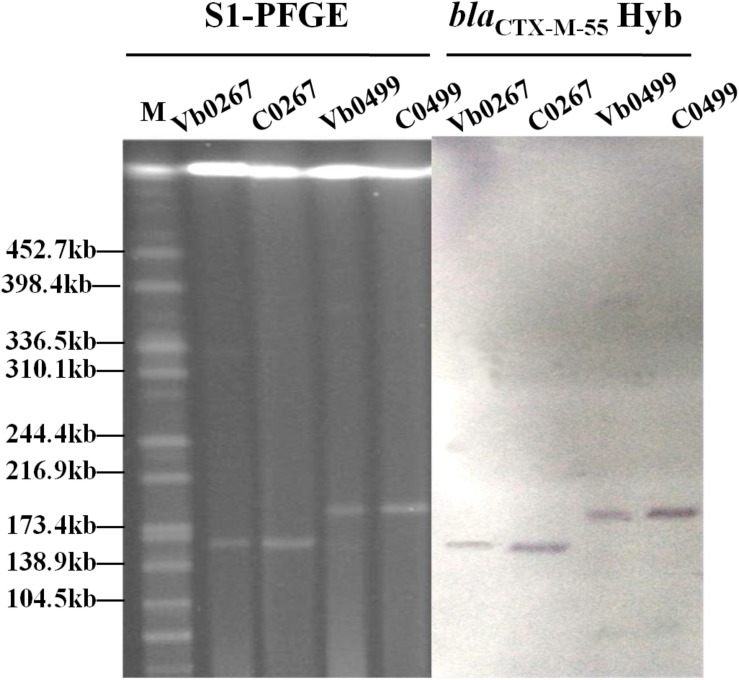
S1-PFGE and Southern hybridization analysis of carriage of *bla*_CTX–M–55_ -bearing plasmids in two *V. parahaemolyticus* strains (Vb0267 and Vb0499) and the corresponding transconjugants (C0267 and C0499). Hyb stands for Southern Hybridization.

A total of 116 *V. parahaemolyticus* strains were isolated from 48 out of the 80 shrimp samples tested. The antimicrobial susceptibility test results revealed that 21 of these 116 *V. parahaemolyticus* strains were resistant to all β-lactam antibiotics tested, including ceftriaxone, cefotaxime, ampicillin and amoxicillin-clavulanic acid (Supplementary Table S1). Carriage of β-lactamase genes in these 21 strains was determined, with results showing that the majority of these strains carried the *bla*_VEB–1_ and *bla*_CMY–2_ genes. These two resistance genes have previously been recovered from strains of the *Vibrio* species and were not included for further analysis in this study (Li et al., 2015a, 2016). Two *V. parahaemolyticus* strains, Vb0267 and Vb0499, were found to carry *bla*_CTX–M–55__,_ which is commonly present in *Enterobacteriaceae* but has never been reported in *Vibrio* species. We therefore focused on deciphering the genetic basis of the evolution of *bla*_CTX–M–55_ in *V. parahaemolyticus*. Conjugation experiments performed on these two strains showed that they could transfer the cephalosporin-resistance phenotype to strain J53 (Table 1). S1-PFGE and Southern hybridization of both parental strains and transconjugants revealed that the *bla*_CTX–M–55_ gene was harbored by plasmids with sizes of ca. 160 and 190 kb, respectively, in the two strains (Figure 1). PFGE fingerprint analysis revealed that Vb0267 and Vb0499 exhibited distinct PFGE profiles (Supplementary Figure S1). Multilocus sequence typing (MLST) analysis showed that both strains belong to unknown genetic types.

**TABLE 1:** MICs of different antibiotics among *Vibrio parahaemolyticus* strains tested in this study and the correspondingtransconjugants.

**Strain ID**	**Species**	**Date of isolation**	**Source of isolation**	**MIC (mg/L)^a^**											**β-lactamase gene**	**β-lactamase-encoding Plasmid (kb)^b^**
		
				**CRO**	**CTX**	**AMC**	**AMP**	**TET**	**AMK**	**GEN**	**CIP**	**NAL**	**CHL**	**SXT**		
J53	*E. coli*			0.015	0.03	4/2	1	0.5	0.03	0.25	0.03	1	2	0.25/4.75		
Vb0267	*V. parahemolyticus*	2015.8.24	Shrimp	>16	>16	8/4	>64	16	8	1	0.25	2	8	2/38	*bla*_CTX–M–55_	∼160 k
C0267	*E. coli*			>16	>16	16/8	>64	16	0.5	0.5	0.03	2	4	0.25/4.75	*bla*_CTX–M–55_	∼160 k
Vb0499	*V. parahemolyticus*	2015.10.26	Shrimp	>16	>16	8/4	>64	8	2	32	0.25	2	4	2/38	*bla*_CTX–M–55_	∼190 k
C0499	*E. coli*			>16	>16	16/8	>64	16	0.5	16	0.25	2	2	0.25/4.75	*bla*_CTX–M–55_	∼190 k

**^a^CRO, ceftriaxone; CTX, cefotaxime; AMC, amoxicillin-clavulanic acid; Amp, ampicillin; TET, tetracycline; AMK, amikacin; GEN, Gentamicin; CIP, ciprofloxacin; NAL, nalidixic acid; CHL, chloramphenicol; SXT, sulfamethoxazole-trimethoprim. ^b^Determined by S1-PFGE.**

### General Features of the *bla*_CTX–M–55_ -Bearing Plasmids pVb0267 and pVb0499

**FIGURE 2:**
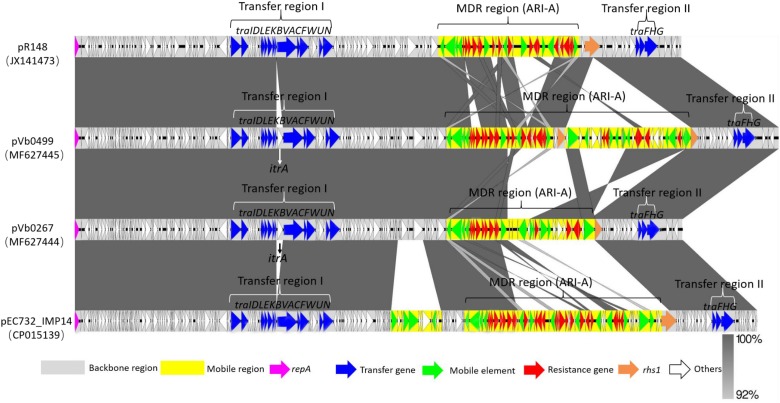
Sequence alignment of type 1a IncC plasmids. Horizontal arrows indicate the location, size and orientation of ORFs. Genes are colored based on their functional classification. Homologous segments generated by a BLASTn comparison are shown as gray blocks that are connected across plasmids. The GenBank accession numbers for each plasmid are as follows: JX141473 (pR148), MF627445 (pVb0499), MF627444 (pVb0267), and CP015139 (pEC732_IMP14).

To investigate the genetic features of the two *bla*_CTX–M–55_-bearing plasmids, the complete sequences of these plasmids were obtained using the Illumina and Nanopore sequencing platforms, and were designated as pVb0267 (MF627444) and pVb0499 (MF627445), respectively. The complete nucleotide sequence of plasmid pVb0267, recovered from strain Vb0267, was 166,467 bp in length, with a mean G+C content of 51.9%, and was found to comprise 183 predicted coding sequences (CDSs). The complete nucleotide sequence of the plasmid pVb0499 was found to be 192,739 bp in size, with an average G+C content of 52.6%, and comprise a total of 213 CDSs. Complete sequence comparison showed that these two plasmids differed mainly by the genetic content of the multidrug-resistant (MDR) region (Figure 2). Sequences outside this region exhibited 100% identity between the two plasmids. Besides, BLAST analysis revealed that the backbones of pVb0267 and pVb0499, after removing the mobile elements and the MDR regions, shared an extremely high degree of genetic similarity (query coverage 98% and identity 99%) with a typical type 1a IncC plasmid, namely pR148 (JX141473), which was previously recovered from a *Aeromonas hydrophila* strain in Thailand (Del Castillo et al., 2013). Consistently, based on the presence or absence of *orf1832/orf1847*, *rhs* (*Rearrangement hotspots*)*1/rhs2*, i1 and i2, which were the key features that distinguish between type 1 and type 2 IncC plasmids (Harmer and Hall, 2015), these two plasmids, pVb0267 and pVb0499, were assigned as type 1a IncC plasmid. pVb0267 and pVb0499, together with the type 1a IncC reference plasmid pR148, were included in subsequent genomic comparison analysis (Figure 2).

The linear sequence comparison revealed two major differences in the backbones of pVb0267, pVb0499, and pR148. First, the *itrA* gene, encoding a Group II intron reverse maturase, was inserted at a site downstream of *traA* in pVb0267 and pVb0499, but not pR148. It should be noted that *itrA* is usually located in a group II intron sequence and barely exists alone (Rodríguez-Martínez et al., 2012). The acquisition mechanism of *itrA* by pVb0267 and pVb0499 was not clear. Second, only the *rhs1* gene of pR148 was intact among these three plasmids. A fragment of N-terminus of the *rhs1* gene was found deleted in pVb0267, whereas a Tn*5403*-mediated transposon was inserted at a site within the *rhs1* gene in pVb0499. These features were not observable in pR148 (Supplementary Figure S2).

Except for the slight genetic differences in the backbones of these three plasmids, the master type 1a IncC plasmid backbone genes (Zhang et al., 2014) involved in replication (*repA*), partitioning and stability (*parAB*, *parM*, and *stbA*) and DNA metabolism (*ssb*–*bet*–*exo*, *ter*–*kfrA*, *int*–*yacC*, *nuc*, *uvrD*, and *pri*), as well as conjugative transfer (*tra*), were shared by the three plasmids (Supplementary Figure S2). The genes involved in plasmid conjugative transfer (*tra*) were separated into two regions (transfer region I and transfer region II) by an MDR region in these three plasmids. The transfer region I, upstream of MDR, was a 30 kb region containing 13 genes (*traIDLEKBVACFWUN*). The remaining three *tra* genes (*traFHG*) were located within another 6 kb region, namely transfer region II (Figure 2).

### Comparison of the MDR Region in Type 1a IncC Plasmids

Various previous studies have reported that the modular structure of each plasmid was discriminated as type 1a IncC backbone, with insertion of one or more separate accessory modules. These accessory modules were further dissected as resistance islands including the IS*Ecp1*-*bla*_CMY_ unit, ARI-B (with the *sul2* gene), and ARI-A (with a class 1 integron) resistance islands as well as the *bla*_KPC–2_ -bearing region, with one or more of these resistance islands inserted at various sites in the backbone of each plasmid (Harmer and Hall, 2014, 2015). Additionally, there appears to be a strong tendency for resistance genes to be incorporated into the *rhs1* gene (Harmer and Hall, 2015). In the case of pVb0267 and pVb0499, ARI-A was detected in these two type 1a IncC plasmids. Moreover, some resistance genes were found inserted into the *rhs1* gene (Supplementary Table S2).

**FIGURE 3:**
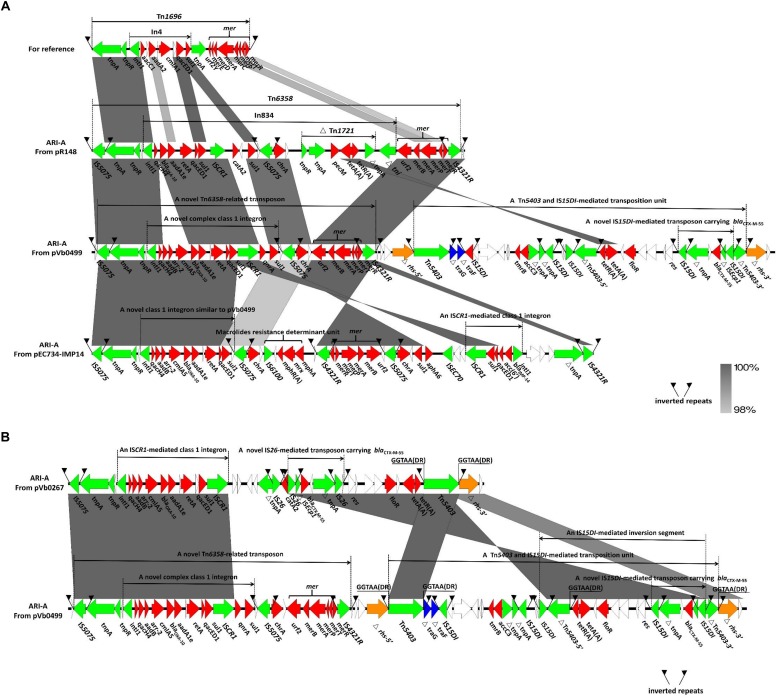
Genetic features of ARI-A islands. **(A)** Comparison of ARI-A islands between pR148, pVb0499, and pEC734-IMP14. Tn1696 are prototype mobile elements used as a reference for sequence comparison, with an accession number of U12338. **(B)** Comparison of ARI-A islands between pVb0267 and pVb0499. Direct repeats (DRs) were produced by independent insertion of Tn*5403*. Genes are denoted by arrows. Genes, mobile elements and other features are colored based on their functional classification. Shading denotes regions of homology (nucleotide identity > 90%).

The structure of the ARI-A region in a typical type 1a IncC plasmid pRMH760 was previously determined and described as being flanked by IS*4321*/ IS*5075* (Sekizuka et al., 2011; Wasyl et al., 2015). A recent study reveals that the ARI-A region in the majority of sequences of type 1a IncC plasmids are located in the same site as that in pRMH760, namely 1711 bp upstream of the *rhs1* gene. Hence the extremities of this region are highly conserved (Harmer and Hall, 2015). Based on the presence of IS*4321*/ IS*5075*, ARI-A islands were identified in pVb0267 and pVb0499. The subsequent ARI-A sequence analysis by BLAST indicated that a plasmid known as pEC732_IMP14 (CP015139), recovered from *Escherichia coli*, exhibited the highest degree of genetic similarity (query coverage 49% and identity 99%) with the ARI-A sequence of pVb0499. Complete ARI-A sequence comparison between pVb0267, pVb0499, pR148, and pEC732_IMP14 was then performed (Figure 3).

Comparative analysis of the ARI-A sequences revealed that all the complex transposon units within the ARI-A region of pVb0267, pVb0499, pR148, and pEC732_IMP14 were derivatives of Tn*1696*, a transposon belonging to the Tn*21* subgroup of the Tn*3* family (Harmer et al., 2014). Tn*1696* was generated from insertion of class 1 integron In*4* at a site within the resolution (*res*) site of a core structure IRL (inverted repeat left)-*tnpA* (transposase)-*tnpR* (resolvase)-*res*-*mer* (mercury resistance locus)-IRR (inverted repeat right) (Figure 3A) (Partridge et al., 2001). The four Tn*1696* derivatives identified in this study differed from the typical Tn*1696* primarily by insertion of a distinct integron or integron-associated regions (Figure 3A). Specifically, the Tn*1696* derivative in pR148 was designated as Tn*6358*, in which a complex class 1 integron (In*834*) harboring two intrinsic resistance regions (the cassette array *qacH4-bla*_OXA–10_*-aadA1e* and *catA2*) and two additional integrated resistance regions [the *chrA* region and a truncated Tn*1721* carrying the class A tetracycline module *tetA*(A)-*tetR*(A) (Kim et al., 2008)], was inserted into the *res* gene (Allmeier et al., 1992). Compared to In*834*, a novel complex class 1 integron was observed in pVb0499 (Figure 3A). In the cassette array (*qacH4-bla*_OXA–10_*-aadA1e*), three additional resistance genes (*aadB, arr-2*, and *cmlA5*) were found to be located between *qacH4* and *bla*_OXA–10_, resulting in a new cassette array (*qacH4-aadB-arr-2-cmlA5-bla*_OXA–10_*-aadA1e*). Besides, the *catA2* region was replaced by the *qnrA* gene, which is responsible for quinolones resistance; this novel complex class 1 integron lacked the truncated Tn*1721* carrying *tetA*(A)-*tetR*(A).

In the ARI-A of pEC732_IMP14, a macrolide resistance determinant unit (IS*6100*-*mphR*(A)-*mrx*-*mph*(A)) was found embedded at the upstream region of the *mer* module. Moreover, a class 1 integron harboring the cassette array (*bla*_IMP–14_-*acc* (6′)-*qacED1*) was found to be located near the right end of ARI-A. However, the *qnrA* gene was not found in pEC732_IMP14. It should be noted that the orientation of the *chrA* and *mer* segments was opposite to each other in pEC732_IMP14 and pVb0499, presumably due to homologous recombination events that occurred in ARI-A. Compared to the ARI-A in pVb0499, the one in pVb0267 has a 11940-bp deletion that spans a region from IS*CR1* to IS*4321R*, a recognized right end region of ARI-A (Figure 3B). In other words, the ARI-A in pVb0267 has lost the *qnrA* gene, the *chrA* region, the *mer* module and IS*4321R*. The deletion events such as those occurred in pVb0267 were also observed in other type 1 IncC plasmids, which were usually associated with insertion of mobile elements such as IS*26* (Lin et al., 1984; Partridge et al., 2011; Letchumanan et al., 2015a).

Besides being located in ARI-A, some resistance genes such as *bla*_CTX–M–55_ were also found inserted in the *rhs1* gene of both pVb0267 and pVb0499. An *rhs* gene was first identified as the site that promoted recombination in *Escherichia coli* (Lin et al., 1984). Replacement of part of the C-terminus of *rhs* is one of the key features that distinguish between type 1 and type 2 IncC plasmids (Harmer and Hall, 2014). In particular, detailed analysis of the *rhs1* region in pVb0267 showed that a deletion of the *rhs*-5′ region occurred in pVb0267 due to the insertion of mobile elements like Tn*5403* and IS*26*, which also led to the absence of the right end of ARI-A in pVb0267 as described above (Figure 3B). In the case of pVb0499, a novel composite transposon, comprising two complex transposon Tn*5403* at both ends, was found in the *rhs1* gene, spliting *rhs1* into two parts, namely △*rhs*-5′ and△*rhs*-3′ (Figure 3B). Analysis of the novel composite transposon revealed that the two Tn*5403* elements were flanked by two typical paired 50 bp inverted repeats (IRL and IRR), which were in turn flanked by two 5 bp direct repeats (GGTAA) (DRs; target site duplication signals for transposition), suggesting that this composite transposon was acquired by the pVb0499 backbone via transposition event (Figure 3B). Remarkably, a 18,607 bp highly conserved sequence segment flanked by two copies of IS*26* or IS*15DI* (variant of I*S26*) was found within the *rhs1* region in pVb0267 and pVb0499. Nevertheless, the orientation of the conserved segment was opposite to each other between pVb0267 and pVb0499. Due to the inversion which occurred in pVb0499, the Tn*5403* at the right end of the composite transposon was spilted into two parts, namely △Tn*5403*-5′ and △Tn*5403*-3′, which might in turn lead to the loss of ability to transpose independently (Figure 3B). Altogether, these evidences suggest that intramolecular homologous recombination mediated by IS*26*/IS*15DI* occurred during the process of evolution of the MDR region of plasmids pVb0267 and pVb0499.

### Comparison of the Genetic Contexts of *bla*_CTX–M–55_ in Vb0267, Vb0499 and Other Gram-Negative Isolates

**FIGURE 4:**
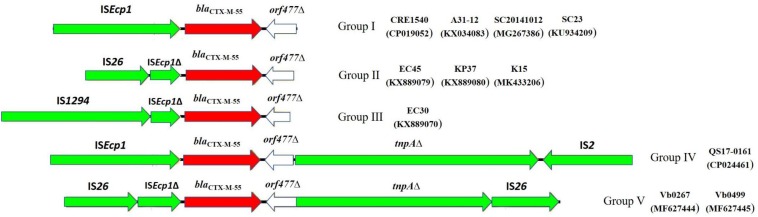
The surrounding regions of *bla*_CTX–M–55_ gene in this study. Group I (IS*Ecp1*-*bla*_CTX–M–55_-*orf477*Δ) was found in isolates (A31-12, CRE1540, SC20141012, SC23); Group II (IS*26*-IS*Ecp1*Δ-*bla*_CTX–M–55_-*orf477*Δ) was found in isolates (EC45, KP37,K15); Group III (IS*1294*-IS*Ecp1*Δ-*bla*_CTX–M–55_-*orf477*Δ) was found in isolate (EC30); Group IV (IS*Ecp1*-*bla*_CTX–M–55_-*orf477*Δ-*tnpA*Δ-IS*2*) was found in isolate (QS17-0161); Group V (IS*26*-IS*Ecp1*-*bla*_CTX–M–55_-*orf477*Δ -*tnpA*Δ-IS*26*) was found in isolates (Vb0267, Vb0499).

To obtain a deeper understanding of the transmission and dissemination of *bla*_CTX–M–55_ gene in different genetic environments, nine *bla*_CTX–M–55_-positive isolates of *Enterobacteriaceae* identified in recent years, together with Vb0267 and Vb0499, were included in a genomic comparison of the flanking region of *bla*_CTX–M–55_. Among these isolates, four were *Escherichia coli*, four were *Klebsiella pneumoniae* and one *Salmonella enterica*. The result of comparison is presented in Figure 4. Generally, all the 11 isolates harbored IS*Ecp1* in the region upstream of *bla*_CTX–M–55_ (in five cases, IS*Ecp1* was truncated by IS*26*, and in one case, it was truncated by IS*1294*), whereas *orf477*Δ was detected downstream of the *bla*_CTX–M–55_ gene from all the strains. The 11 *bla*_CTX–M–55_-positive isolates were further divided into five different groups according to the genetic contexts of *bla*_CTX–M–55_ gene, [Group I (4 isolates), Group II (3 isolates), Group III (1 isolate), Group IV (1 isolate), and Group V (2 isolate)]. Group I (IS*Ecp1*-*bla*_CTX–M–55_-*orf477*Δ) was the most common. The arrangement of Group II (IS*26*-IS*Ecp1*Δ-*bla*_CTX–M–55_-*orf477*Δ) and Group III (IS*1294*-IS*Ecp1*Δ-*bla*_CTX–M–55_-*orf477*Δ) was similar with Group I, although IS*Ecp1* was disrupted by IS*26* in Group II and by IS*1294* in Group III. Group IV (IS*Ecp1*-*bla*_CTX–M–55_-*orf477*Δ-*tnpA*Δ-IS*2*) and Group V (IS*26*-IS*Ecp1*-*bla*_CTX–M–55_-*orf477*Δ-*tnpA*Δ-IS*26*) both harbored *tnpA* downstream of *orf477*Δ, while the *tnpA* gene was truncated by IS*2* in Group IV and by IS*26* in Group V.

## DISCUSSION

The continuous and extensive use of antibiotics in the aquaculture industry facilitates the development of various resistant isolates and the dissemination of resistance genes within the bacterial population in the environment (Tendencia and Peña, 2002; Rebouças et al., 2011). All the antibiotics used in this study are recommended in the treatment of infections caused by strains of the *Vibrio* sp., including tetracycline, cefotaxime, amikacin, gentamicin, and trimethoprim-sulfamethoxazole, some of which, tetracycline and chloramphenicol in particular, are also widely used in aquaculture industry (Shaw et al., 2014; Letchumanan et al., 2015b). In this study, 82.8% of the *V. parahaemolyticus* isolates recovered from shrimp samples exhibited resistance to ampicillin. Our results are in agreement with other studies that reported resistance to ampicillin among the *V. parahaemolyticus* isolates recovered from seafood samples (Letchumanan et al., 2015b; Li et al., 2015b). Due to the extensive use of first generation antibiotics including ampicillin, the efficacy of ampicillin for *Vibrio* treatment has decreased (Letchumanan et al., 2015b).

Resistance to the third generation cephalosporins was observed in our *V. parahaemolyticus* isolates, with 18% of the test isolates being resistant to cefotaxime and 19% being resistant to ceftriaxone. This rate is much lower than to that reported in other studies on the resistance to third generation cephalosporin among *V. parahaemolyticus* isolates, with the rate of resistance to cefotaxime typically in the range of 73–80% (Sahilah et al., 2014; Letchumanan et al., 2015b). The discrepancies regarding the resistance phenotype to third generation cephalosporin could possibly due to the difference in test methodology or geographical variation.

**FIGURE 5:**
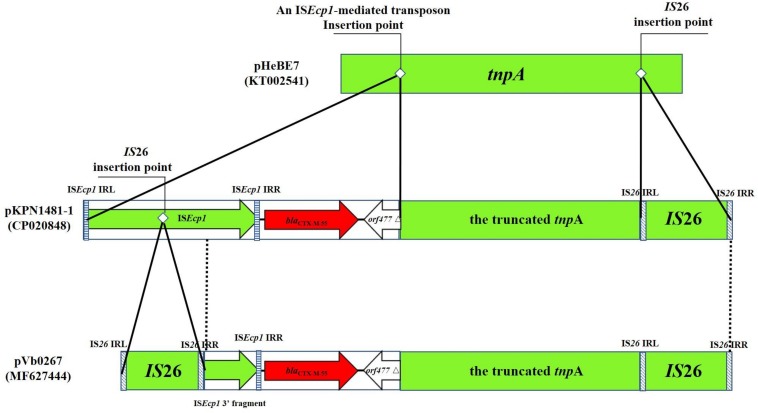
Proposed mechanism of formation of a novel IS*26*-mediated transposon carrying the *bla*_CTX–M–55_ gene. The transposon and insertion sequence (IS) are indicated by rectangles. Genes harbored in an IS*Ecp1*-mediated transposon are denoted by arrows. Dotted vertical lines indicate the region of homology between pKPN1481-1 and pVb0267. Striped rectangles indicate the IRs of IS*26* and IS*Ecp1*, respectively. The insertion point of IS*26* and an IS*Ecp1*-mediated transposon are indicated by white rhombi. pHeBE7 and pKPN1481-1 are references for sequence comparison, with accession numbers being KT002541 and CP020848, respectively.

pVb0267 and pVb0499 are the first two sequenced *bla*_CTX–M–55_-bearing type 1a IncC plasmids recovered from non-clinical isolates in China. Analysis of their structures provides new insight into the plasticity of the genetic context of ARI-A and the role of IS*26*/IS*15DI* in mediating homologous recombination events that occurred in *rhs* region, as well as mobilization of the *bla*_CTX–M–55_-containing transposon in pVb0267 and pVb0499. In the *rhs* resistance region of pVb0267 and pVb0499, a highly conservative fragment harboring *bla*_CTX–M–55_ was observed (Figure 3B). The complex fragment is composed of the IS*Ecp1* element, the *bla*_CTX–M–55_ gene, a *orf477* truncated element (*orf477*△) and a truncated *tnpA* gene. Interestingly, the cassette array was flanked by two IS*26* or IS*15DI* elements in both pVb0267 and pVb0499. Coincidentally, this arrangement is a representative composite transposon structure, suggesting that this cassette array has the potential to undergo horizontal transfer (Kidwell, 2005). However, sequence analysis did not reveal the presence of any distinct direct repeats (DRs). This finding is intriguing. Likewise, the underlying mechanism by which *bla*_CTX–M–55_ was acquired by pVb0267 and pVb0499 remains to be determined. To test a possible mechanism of formation of this apparent composite transposon, further sequence alignment by BLASTn against the transposon in pVb0267 was performed. Two similar sequences were found. One was from pA31-12 (KX034083) and the other was from pKPN1481-1 (CP020848). All of the three segments were found to share a highly conservative region (99% identity) of 1819 bp in size, and contain an IS*Ecp1* element, the *bla*_CTX–M–55_ gene and the *orf477*△ element (Figure 5). Taken together, we hypothesize that at the initial stage of evolution, a copy of IS*26* and an IS*Ecp1*-mediated transposon carrying *bla*_CTX–M–55_ and *orf477*△ was inserted into a *tnpA* gene, resulting in formation of a similar structure found in pKPN1481-1. Another IS*26* was embedded in the IS*Ecp1*, which eventually formed in the composite transposon observed in pVb0267. However, the absence of direct repeats may result from subsequent IS*26*-mediated adjacent deletions or a conservative IS*26*-mediated cointegrate formation event (Harmer et al., 2014). It is necessary to further assess the mobility of IS*26* or its variant (Figure 5).

More importantly, the *bla*_CTX–M_ family extended-spectrum β-lactamase-encoding genes have been widely disseminated among cephalosporin resistant clinical strains of *Enterobacteriaceae* such as those of *Escherichia coli* and *K. pneumoniae* (Zhang et al., 2014). In contrast, resistance toward cephalosporins in foodborne pathogenic *Vibrio* spp. remains relatively rare (Li et al., 2015a). To our knowledge, this is the first report of recovery of *bla*_CTX–M–55_ from *Vibrio* spp. These findings indicated that cephalosporin resistance mediated by the *bla*_CTX–M_ and other related resistance determinants in *Vibrio parahaemolyticus* may become a major concern not only due to its growing clinical relevance as a key foodborne pathogen, but also because *Vibrio parahaemolyticus* may serve as a *bla*_CTX–M–55_ reservoir from which the resistance element may be readily transferred to other pathogenic organisms. This view has been confirmed by the finding that pVb0267 and pVb0499 could be successfully transferred to the J53 recipient strain in the present study. If no effective measures are taken, these evolutionary characteristics of ESBL-producing foodborne strains of *Vibrio* spp. may complicate future management of antibiotic resistance in China. Further investigations are required to confirm if these plasmids are responsible for causing an increasing incidence of carriage of *bla*_CTX–M–55_ or other resistance elements in strains of the *Vibrio* spp. in China and other countries. This study also indicated that the increasing evolution of conjugative plasmids carrying different antimicrobial resistance gene could be attributed to bacterial adaption to aquaculture environment with increasing amounts of antibiotics.

## Data Availability

The datasets generated for this study can be found in Genbank, MF627444 and MF627445.

## Author Contributions

All authors listed have made a substantial, direct and intellectual contribution to the work, and approved it for publication.

## Conflict of Interest Statement

The authors declare that the research was conducted in the absence of any commercial or financial relationships that could be construed as a potential conflict of interest.
