# Supplementary material for: Genetic Characterization of blaCTX–M–55 -Bearing Plasmids Harbored by Food-Borne Cephalosporin-Resistant Vibrio parahaemolyticus Strains in China
Source: Front Microbiol. 2019 Jun 18;10:1338. doi: 10.3389/fmicb.2019.01338 (PMC6591265; doi:10.3389/fmicb.2019.01338)
Supplement: Supplementary file 1 [file Data_Sheet_1.pdf]

Supplementary materials

Genetic characterization of $bla_{CTX-M-55}$ -bearing plasmids harbored by food-borne cephalosporin-resistant *Vibrio parahaemolyticus* strains in China

Zhiwei Zheng[1,2], Ruichao Li[2,3,4], Lianwei Ye[2,3], Edward Wai-chi Chan[3], Xiaodong Xia[1*], Sheng Chen[2,3*]

[1]College of Food Science and Engineering, Northwest A&F University, Yangling, Shaanxi, P. R. China;

[2]Shenzhen Key Laboratory for Food Biological Safety Control, Food Safety and Technology Research Centre, The Hong Kong PolyUShenzhen Research Institute, Shenzhen, P. R. China;

[3]State Key Laboratory of Chirosciences, Department of Applied Biology and Chemical Technology, The Hong Kong Polytechnic University, Hung Hom, Kowloon, Hong Kong.

[4]College of Veterinary Medicine, Yangzhou University, Yangzhou, Jiangsu, P. R. China.

Running Title: First identification of $bla_{CTX-M-55}$ in *Vibrio* ***parahaemolyticus***

*Corresponding author. Xiaodong Xia, Email: foodscixiaodong@yahoo.com; or Sheng Che, Tel: (852)-3400-8795; Fax: (852)-2364 9932; Email: sheng.chen@polyu.edu.hk

Supplementary Table S1. Antibiotic susceptibility profiles of 21 cephalosporin-resistant *V. parahaemolyticus* strains isolated form shrimp samples.

Strain ID	Species	Source of isolation	Isolation date	MIC(mg/L)[a]										
				CRO	CTX	AMC	AMP	TET	AMK	GEN	CIP	NAL	CHL	SXT
Vb0263	*V. parahaemolyticus*	Shrimp	2015.8.24	8	2	16/8	＞64	2	4	2	0.25	8	4	＞8/152
Vb0264	*V. parahaemolyticus*	Shrimp	2015.8.24	4	2	16/8	＞64	1	4	2	0.25	＞64	2	＞8/152
Vb0265	*V. parahaemolyticus*	Shrimp	2015.8.24	16	＞16	8/4	＞64	4	8	16	0.015	1	2	1/19
Vb0266	*V. parahaemolyticus*	Shrimp	2015.8.24	＞16	＞16	8/4	＞64	1	8	8	0.25	2	8	4/72
Vb0267	*V. parahaemolyticus*	Shrimp	2015.8.24	＞16	＞16	8/4	＞64	16	8	1	0.25	2	8	2/38
Vb0314	*V. parahaemolyticus*	Shrimp	2015.9.14	8	4	32/16	＞64	4	2	2	0.5	2	4	＞8/152
Vb0390	*V. parahaemolyticus*	Shrimp	2015.10.8	＞16	＞16	32/16	＞64	16	4	2	0.5	1	1	＞8/152
Vb0419	*V. parahaemolyticus*	Shrimp	2015.10.12	16	＞16	8/4	＞64	0.5	4	16	0.5	1	1	2/38
Vb0441	*V. parahaemolyticus*	Shrimp	2015.10.19	16	8	4/2	64	0.5	4	8	0.5	1	1	0.5/9.5
Vb0442	*V. parahaemolyticus*	Shrimp	2015.10.19	16	8	4/2	64	0.5	4	8	0.25	1	0.5	0.5/9.5
Vb0448	*V. parahaemolyticus*	Shrimp	2015.10.19	16	16	4/2	64	0.5	4	8	0.5	1	0.5	0.25/4.75
Vb0472	*V. parahaemolyticus*	Shrimp	2015.10.26	16	16	4/2	64	0.5	8	8	0.5	2	1	0.25/4.75
Vb0473	*V. parahaemolyticus*	Shrimp	2015.10.26	16	4	8/4	＞64	0.5	4	1	0.5	2	1	0.5/9.5
Vb0481	*V. parahaemolyticus*	Shrimp	2015.10.26	16	16	4/2	64	0.25	4	8	0.5	1	1	0.5/9.5

Vb0492	*V. parahaemolyticus*	Shrimp	2015.10.26	>16	>16	32/16	>64	8	2	1	0.25	2	4	>8/152
Vb0494	*V. parahaemolyticus*	Shrimp	2015.10.26	>16	>16	8/4	>64	0.5	4	8	0.25	1	0.5	0.5/9.5
Vb0495	*V. parahaemolyticus*	Shrimp	2015.10.26	>16	>16	32/16	>64	8	2	1	0.25	1	4	>8/152
Vb0496	*V. parahaemolyticus*	Shrimp	2015.10.26	>16	>16	8/4	>64	0.5	2	16	0.25	2	0.5	1/19
Vb0497	*V. parahaemolyticus*	Shrimp	2015.10.26	>16	>16	32/16	>64	8	2	1	0.25	2	4	>8/152
Vb0498	*V. parahaemolyticus*	Shrimp	2015.10.26	>16	>16	32/16	>64	8	2	1	0.25	2	4	>8/152
Vb0499	*V. parahaemolyticus*	Shrimp	2015.10.26	>16	>16	8/4	>64	8	2	32	0.25	2	4	2/38

[a]CRO, ceftriaxone; CTX, cefotaxime; AMC, amoxocillin-clavulanic acid; Amp, ampicillin; TET, tetracycline; AMK, amikacin; GEN, Gentamicin; CIP, ciprofloxacin; NAL, nalidixic acid; CHL, chloramphenicol; SXT, sulfamethoxazole-trimethoprim.

Supplementary Table S2. Resistance genes harboured by plasmids tested in this work.

Plasmid	Resist. Genes	Resistance phenotype	Resist. Genes	Resistance phenotype
pR148	*qacH*	Quaternary ammonium compound resistance	*catA2*	Phenicol resistance
	bla$_{\text{OXA-10}}$	β-lactam resistance	*chrA*	Chromate resistance
	aadA1	Aminoglycoside resistance	*tetA(A)*	Tetracycline resistance
	*qacE*Δ*1*	Quaternary ammonium compound resistance	*mer locus*	Mercuric resistance
	sul1	Sulfonamide resistance		
pVb0267	*qacH*	Quaternary ammonium compound resistance	*sul1*	Sulfonamide resistance
	aadB	Aminoglycoside resistance	*catA2*	Phenicol resistance
	arr-2	rifampin resistance	*bla*$_{\text{CTX-M-55}}$	β-lactam resistance
	cmlA5	Chloramphenicol resistance	*floR*	Phenicol resistance
	bla$_{\text{OXA-10}}$	β-lactam resistance	*tetA*(A)	Tetracycline resistance
	aadA1	Aminoglycoside resistance	*mer locus*	Mercuric resistance
	*qacE*Δ*1*	Quaternary ammonium compound resistance		
pVb0499	*qacH*	Quaternary ammonium compound resistance	*qnrA*	Quinolone resistance
	aadB	Aminoglycoside resistance	*chrA*	Chromate resistance
	arr-2	rifampin resistance	*mer locus*	Mercuric resistance
	cmlA5	Chloramphenicol resistance	*tmrB*	tunicamycin resistance
	bla$_{\text{OXA-10}}$	β-lactam resistance	*aacC3*	Aminoglycoside resistance
	aadA1	Aminoglycoside resistance	*tetA*(A)	Tetracycline resistance
	*qacE*Δ*1*	Quaternary ammonium compound resistance	*floR*	Phenicol resistance
	sul1	Sulfonamide resistance	*bla*$_{\text{CTX-M-55}}$	β-lactam resistance
pEC734-IMP14	*qacH*	Quaternary ammonium compound resistance	*sul1*	Sulfonamide resistance
	aadB	Aminoglycoside resistance	*chrA*	Chromate resistance
	arr-2	rifampin resistance	*mph(A)*	Macrolide resistance
	cmlA5	Chloramphenicol resistance	*mer locus*	Mercuric resistance
	bla$_{\text{OXA-10}}$	β-lactam resistance	*aphA6*	Aminoglycoside resistance
	aadA1	Aminoglycoside resistance	*aac(6')*	Aminoglycoside resistance
	*qacE*Δ*1*	Quaternary ammonium compound resistance	*bla*$_{\text{IMP-14}}$	β-lactam resistance

Supplementary Table S3. Group-specific primers used for the assays

PCR name	b-Lactamase(s) targeted	Primer name	Sequence (5 '– 3 ')
Multiplex I TEM, SHV and OXA-1-like	TEM variants including TEM-1 and TEM-2	MultiTSO-_for	CATTTCCGTGTCGCCCTTATTC
		MultiTSO-_rev	CGTTCATCCATAGTTGCCTGAC
	SHV variants including SHV-1	MultiTSO-S_for	AGCCGCTTGAGCAAATTAAAC
		MultiTSO-S_rev	ATCCCGCAGATAAATCACCAC
	OXA-1, OXA-4 and OXA-30	MultiTSO-O_for	GGCACCAGATTCAACTTTCAAG
		MultiTSO-O_rev	GACCCCAAGTTTCCTGTAAGTG
Multiplex II CTX-M group 1, group 2 and group 9	variants of CTX-M group 1 including CTX-M-1, CTX-M-3 and CTX-M-15	MultiCTXMGp1_for	TTAGGAARTGTGCCGCTGYA[a]
		MultiCTXMGp1-2_rev	CGATATCGTTGGTGGTRCCAT[a]
	variants of CTX-M group 2 including CTX-M-2	MultiCTXMGp2_for	CGTTAACGGCACGATGAC
		MultiCTXMGp1-2_rev	CGATATCGTTGGTGGTRCCAT[a]
	variants of CTX-M group 9 including CTX-M-9 and CTX-M-14	MultiCTXMGp9_for	TCAAGCCTGCCGATCTGGT
		MultiCTXMGp9_rev	TGATTCTCGCCGCTGAAG
CTX-M group 8/25	CTX-M-8, CTX-M-25, CTX-M-26 and CTX-M-39 to CTX-M-41	CTX-Mg8/25_for	AACRCRCAGACGCTCTAC[a]
		CTX-Mg8/25_rev	TCGAGCCGGAASGTGTYAT[a]
Multiplex III ACC, FOX, MOX, DHA, CIT and EBC	ACC-1 and ACC-2	MultiCaseACC_for	CACCTCCAGCGACTTGTTAC
		MultiCaseACC_rev	GTTAGCCAGCATCACGATCC
	FOX-1 to FOX-5	MultiCaseFOX_for	CTACAGTGCGGGTGGTTT
		MultiCaseFOX_rev	CTATTTGCGGCCAGGTGA
	MOX-1, MOX-2, CMY-1, CMY-8 to CMY-11 and CMY-19	MultiCaseMOX_for	GCAACAACGACAATCCATCCT
		MultiCaseMOX_rev	GGGATAGGCGTAACTCTCCCAA
	DHA-1 and DHA-2	MultiCaseDHA_for	TGATGGCACAGCAGGATATTC
		MultiCaseDHA_rev	GCTTTGACTCTTTCGGTATTCG
	LAT-1 to LAT-3, BIL-1, CMY-2 to CMY-7, CMY-12 to CMY-18 and CMY-21 to CMY-23	MultiCaseCIT_for	CGAAGAGGCAATGACCAGAC
		MultiCaseCIT_rev	ACGGACAGGGTTAGGATAGY[a]
	ACT-1 and MIR-1	MultiCaseEBC_for	CGGTAAAGCCGATGTTGCG
		MultiCaseEBC_rev	AGCCTAACCCCTGATACA
Multiplex IV VEB, PER and GES	GES-1 to GES-9 and GES-11	MultiGES_for	AGTCGGCTAGACCGGAAAG
		MultiGES_rev	TTTGTCCGTGCTCAGGAT
	PER-1 and PER-3	MultiPER_for	GCTCCGATAATGAAAGCGT
		MultiPER_rev	TTCGGCTTGACTCGGCTGA
	VEB-1 to VEB-6	MultiVEB_for	CATTTCCCGATGCAAAGCGT
		MultiVEB_rev	CGAAGTTTCTTTGGACTCTG
Multiplex V GES and OXA-48-like	GES-1 to GES-9 and GES-11	MultiGES_for	AGTCGGCTAGACCGGAAAG
		MultiGES_rev	TTTGTCCGTGCTCAGGAT
	OXA-48-like	MultiOXA-48_for	GCTTGATCGCCCTCGATT
		MultiOXA-48_rev	GATTTGCTCCGTGGCCGAAA
Multiplex VI IMP, VIM and KPC	IMP variants except IMP-9, IMP-16, IMP-18, IMP-22 and IMP-25	MultiIMP_for	TTGACACTCCATTTACDG[a]
		MultiIMP_rev	GATYGAGAATTAAGCCACYCT[a]
	VIM variants including VIM-1 and VIM-2	MultiVIM_for	GATGGTGTTTGGTCGCATA
		MultiVIM_rev	CGAATGCGCAGCACCAG
	KPC-1 to KPC-5	MultiKPC_for	CATTCAAGGGCTTTCTTGCTGC
		MultiKPC_rev	ACGACGGCATAGTCATTTGC

[a]Y=T or C; R=A or G; S=G or C; D=A or G or T.

Supplementary Figure S1. PFGE profiles of 15 cephalosporin-resistant *V. parahaemolyticus* strains.

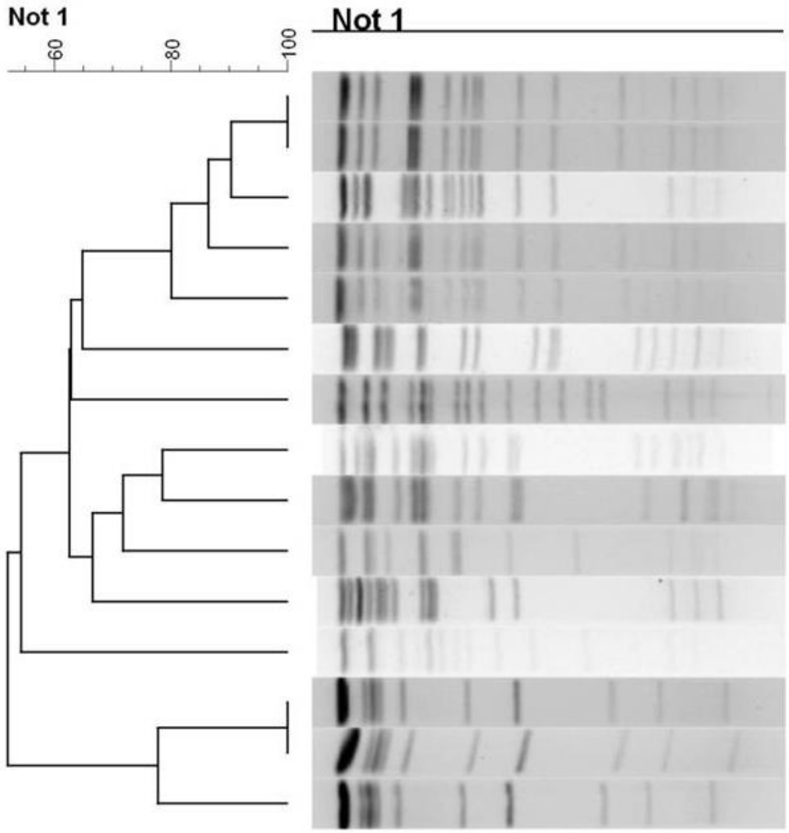

Strain ID	Isolation data	Species	Source of isolation
Vb441	2015.10.19	V.parahemolyticus	shrimp
Vb448	2015.10.19	V.parahemolyticus	shrimp
Vb267	2015.8.24	V.parahemolyticus	shrimp
Vb472	2015.10.26	V.parahemolyticus	shrimp
Vb481	2015.10.26	V.parahemolyticus	shrimp
Vb390	2015.10.8	V.parahemolyticus	shrimp
Vb499	2015.10.26	V.parahemolyticus	shrimp
Vb264	2015.8.24	V.parahemolyticus	shrimp
Vb494	2015.10.26	V.parahemolyticus	shrimp
Vb496	2015.10.26	V.parahemolyticus	shrimp
Vb266	2015.8.24	V.parahemolyticus	shrimp
Vb265	2015.8.24	V.parahemolyticus	shrimp
Vb492	2015.10.26	V.parahemolyticus	shrimp
Vb495	2015.10.26	V.parahemolyticus	shrimp
Vb497	2015.10.26	V.parahemolyticus	shrimp

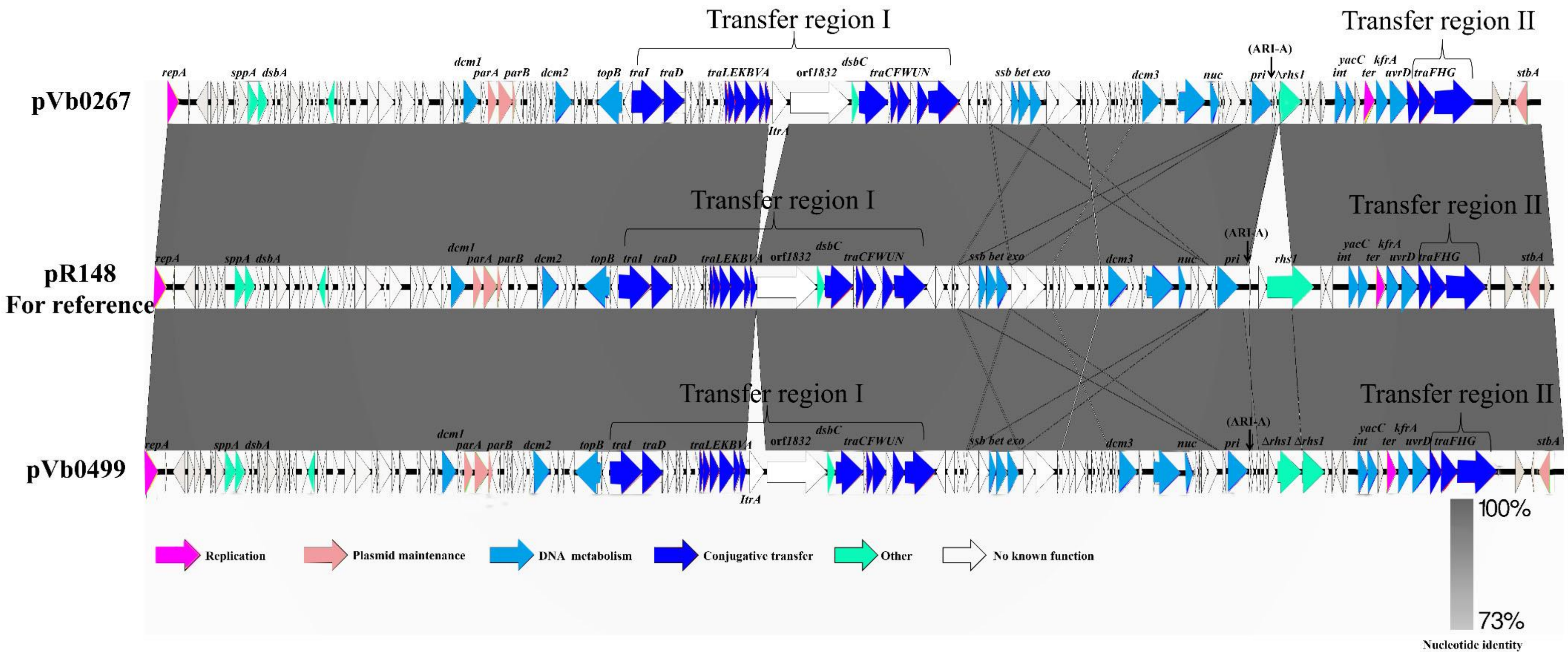

Supplementary Figure S2. Comparative analysis of the plasmid backbones of pR148, pVb0267 and pVb0499. The type1 IncC backbone sequence of pR148 (GenBank accession number JX141473), as defined by Harmer and Hall (2014) was aligned for these three plasmids. Horizontal arrows indicate the location, size and orientation of ORFs.
